# Innovations in Diabetic Macular Edema Management: A Comprehensive Review of Automated Quantification and Anti-vascular Endothelial Growth Factor Intervention

**DOI:** 10.7759/cureus.54752

**Published:** 2024-02-23

**Authors:** Soumya Sharma, Sachin Daigavane, Pranaykumar Shinde

**Affiliations:** 1 Ophthalmology, Jawaharlal Nehru Medical College, Datta Meghe Institute of Higher Education and Research, Wardha, IND

**Keywords:** innovation in ophthalmology, diabetic retinopathy, precision medicine, anti-vegf intervention, automated quantification, diabetic macular edema (dme)

## Abstract

Diabetic macular edema (DME) poses a significant threat to the vision and quality of life of individuals with diabetes. This comprehensive review explores recent advancements in DME management, focusing on integrating automated quantification techniques and anti-vascular endothelial growth factor (anti-VEGF) interventions. The review begins with an overview of DME, emphasizing its prevalence, impact on diabetic patients, and current challenges in management. It then delves into the potential of automated quantification, leveraging machine learning and artificial intelligence to improve early detection and monitoring. Concurrently, the role of anti-VEGF therapies in addressing the underlying vascular abnormalities in DME is scrutinized. The review synthesizes vital findings, highlighting the implications for the future of DME management. Promising outcomes from recent clinical trials and case studies are discussed, providing insights into the evolving landscape of personalized medicine approaches. The conclusion underscores the transformative potential of these innovations, calling for continued research, collaboration, and integration of these advancements into clinical practice. This review aims to serve as a roadmap for researchers, clinicians, and industry stakeholders, fostering a collective effort to enhance the precision and efficacy of DME management.

## Introduction and background

Diabetic macular edema (DME), a complication stemming from diabetic retinopathy (DR), manifests as fluid accumulation in the macula - a critical area responsible for central vision. The macula's edema leads to impairment and metamorphopsia of central vision, often progressing to severe visual loss if left untreated. Understanding the pathophysiology of DME is crucial for appreciating the complexity of its management [1]. The significance of DME extends beyond its immediate impact on visual acuity. Individuals with diabetes face an elevated risk of developing DR, with DME representing a leading cause of vision impairment in this population. The socio-economic implications of vision loss further underscore the importance of effective management strategies. Exploring the broader impact on the quality of life for diabetic patients will provide a context for evaluating the advancements discussed in subsequent sections [2].

Despite advancements in medical technology and therapeutic approaches, managing DME remains a complex and evolving challenge. Factors such as late-stage detection, variability in treatment response, and the need for frequent interventions contribute to the intricate nature of DME management. A comprehensive understanding of these challenges sets the stage for exploring innovative solutions and interventions that aim to address these issues [3]. Notably, diabetes is the primary cause of new cases of blindness in the United States. The primary objective of this review is to critically assess recent innovations in DME management, with a specific focus on integrating automated quantification techniques and anti-vascular endothelial growth factor (anti-VEGF) interventions. By examining the latest advancements in diagnostic tools and therapeutic strategies, this review aims to provide insights into the potential improvements in precision, efficiency, and patient outcomes. By synthesizing current literature and clinical findings, we seek to contribute to the ongoing dialogue surrounding the optimization of DME management, fostering a deeper understanding of the challenges and opportunities in this evolving field.

## Review

DME: pathophysiology and clinical manifestations

Pathophysiology of DME

DME is characterized by the abnormal accumulation of excess fluid in the macula resulting from microvascular changes in the retina. These changes compromise the integrity of the blood-retinal barrier, leading to the leakage of plasma constituents into the surrounding retina and causing retinal edema [4]. The pathophysiology of DME involves the breakdown of the inner and outer blood-retinal barriers, pericyte loss, breakdown of endothelial cell-cell junctions, and the chronic accumulation of advanced glycated end products (AGEs) related to hyperglycemia [5-7]. This disruption in the blood-retinal barrier results in fluid leakage into the macula, leading to the distinctive cystic retinal thickening or lipid deposition observed in DME [6]. DME stands as a significant contributor to vision loss in individuals with diabetes. Its pathogenesis is not solely attributed to vascular endothelial growth factor (VEGF) but also involves inflammatory processes and dysfunction in the neurovascular unit [7]. Clinical manifestations of DME encompass retinal thickening, hard exudates, and decreased visual acuity. Effectively managing DME requires a multifaceted approach, including rigorous control of diabetes, blood glucose, hypertension, and dyslipidemia are known to be important risk factors for DME. Anti-VEGF therapy, alongside exploration of novel therapeutic targets, constitutes integral components of this management strategy [7,8].

Clinical Symptoms and Manifestations

DME gives rise to a spectrum of visual disturbances, manifesting in various ways. First and foremost, the accumulation of excess fluid, swelling, and thickening in the macula induces visual distortions, causing objects to appear distorted or blurred, often adopting a waviness that challenges the clarity of vision [9]. This fluid-related impairment extends to a broader impact on vision, leading to blurry vision, double vision, difficulty discerning colors, and the perception of dark spots [10]. Moreover, DME exerts a significant toll on visual acuity, diminishing central vision and impairing the ability to perceive fine details. This, in turn, poses challenges in everyday activities such as reading and driving [11]. The retinal changes associated with DME are observable through a comprehensive eye examination, with the accumulation of excess fluid in the macula leading to discernible retinal thickening [10]. Notably, the intricate relationship between DME and DR underscores the gravity of its association with diabetes. Poor glycemic control and the duration of diabetes emerge as prominent risk factors for the development of DME, emphasizing the critical role of diabetes management in mitigating these vision-related complications [8].

Impact on Vision and Quality of Life

DME emerges as a consequential complication of diabetes mellitus, significantly impacting the visual function of individuals in their working-age years and consequently influencing their overall quality of life [12]. The assessment of DME's effect on vision and quality of life involves various tools and studies, exemplified by Fenwick et al.'s investigation into the psychometric properties of DR and DME quality-of-life item banks utilizing computerized adaptive testing (CAT) [13]. This study found that the item pools exhibited satisfactory psychometric properties, and CAT simulations determined the average number of items administered at high and moderate precision levels [13]. In a parallel study, Fenwick et al. explored the impact of DR and DME on health-related quality of life (HRQOL) in patients with type 1 and type 2 diabetes, revealing that the presence or severity of DR/DME, coupled with concomitant vision loss, significantly influenced HRQOL [14]. Another investigation by Kojima et al. delved into the repercussions of DME on vision quality and vision-specific quality of life among type 2 diabetic patients. Utilizing the 25-item mean VFQ-25 subscale scores, the study determined that type 2 diabetes patients with macular edema experienced a diminished visual-related quality of life (VR-QOL) compared to those with type 1 glaucoma or cataracts. However, their VR-QOL was similar to individuals with age-related macular degeneration (ARMD) [15]. Further shedding light on the socioeconomic impact, the Canadian Burden of Diabetic Macular Edema Observational Study (C-REALITY) highlighted substantial DME-related costs in the Canadian healthcare system. Notably, these costs increased with the severity of visual acuity loss, coinciding with a decline in vision-related quality of life [16].

Traditional diagnostic and monitoring approaches

Fundus Photography and Optical Coherence Tomography

Fundus photography is a rapid and non-invasive technique, offering a 30- to 50-degree view of the retina and optic nerve. This method is crucial in facilitating early and accurate diagnosis of retinal abnormalities, enabling timely treatment and improved therapeutic outcomes. However, its utility has limitations. While fundus photography is adept at providing an overall view, it needs to thoroughly examine the retinal layers and quantify changes in retinal structure over time. Additionally, its dependence on accurate color representation introduces susceptibility to artifacts and variations in image quality [17,18]. In contrast, optical coherence tomography (OCT) emerges as a powerful diagnostic tool, providing a meticulous, cross-sectional perspective of the retina. This capability facilitates the examination of retinal layers and the quantification of structural changes over time. OCT proves particularly beneficial in diagnosing and monitoring conditions such as DME, ARMD, and glaucoma. Notably, OCT surpasses fundus photography in glaucoma screening by enabling the precise quantification of retinal structures, thereby enhancing diagnostic accuracy [18,19]. Recognizing the complementary strengths of both techniques is crucial in ophthalmic practice. Fundus photography captures an overall view of the retina and optic nerve, offering a broad perspective. Conversely, OCT is indispensable for detailed, cross-sectional examinations and quantifying subtle retinal changes. The choice between these techniques hinges on the specific clinical context and the requisite information for accurate diagnosis and monitoring. Ultimately, the judicious integration of fundus photography and OCT enhances the diagnostic capabilities in ophthalmology, providing a comprehensive approach to patient care.

Limitations of Manual Quantification

The manual quantification of DME through traditional methods presents several inherent limitations. Although manual grading by reading centers has been the standard in clinical trials, doubts have arisen regarding the efficacy of reading center error correction in automated OCT retinal thickness measurements in eyes afflicted with DME. A study published in Investigative Ophthalmology & Visual Science scrutinized the value of reading center error correction in automated OCT retinal thickness measurements for DME, concluding that correcting automated errors had negligible effects on interpreting results from DME clinical trials, provided adequate quality control measures were in place [20]. Moreover, the precision of manual correction is contingent on the operator, and images with segmentation errors may necessitate manual correction, introducing potential variability in the results [21]. In contrast, the application of artificial intelligence (AI) in precision medicine for DME demonstrates promise in automating the quantification of macular fluid in retinal diseases and assessing their response to anti-VEGF therapy. This approach seeks to surmount the limitations of manual quantification by mitigating operator-dependent variability and enhancing the speed, precision, accuracy, and reproducibility of quantifying retinal pathology [22]. The shortcomings of manual quantification in DME, including operator-dependent variability and the need for manual correction of segmentation errors, prompt the exploration of alternative approaches. Integrating AI and automated quantification methods aims to address these limitations, ultimately enhancing the accuracy and reproducibility of DME quantification. This shift toward automated techniques holds significant promise for advancing the precision and efficiency of DME management in clinical settings.

Challenges in Early Detection and Monitoring

The challenges inherent in the early detection and monitoring of DME underscore the critical need for precise and reproducible quantification of retinal pathology. Traditional manual methods for quantifying DME may need improved speed, precision, accuracy, and reproducibility. Such challenges can impede the effective early detection and monitoring of DME, introducing variability and potential constraints in clinical assessments [23]. Recognizing these limitations, digital imaging and automated quantification methods have been proposed, particularly those driven by AI. The adoption of digital imaging and automated quantification, leveraging the capabilities of AI, is positioned as a solution to overcome the drawbacks associated with manual techniques. These advanced methods promise to outperform manual quantification in speed, precision, accuracy, and reproducibility. The overarching goal is to enhance the early detection and monitoring of DME, thereby elevating the standard of patient care and optimizing treatment outcomes [23]. Integrating these innovative approaches into clinical practice represents a pivotal step towards addressing the challenges in DME management and ushering in a new era of efficiency and precision in ophthalmic care.

Automated quantification techniques

Advancements in the quantification of retinal ischemia have been made with the imaging modalities of fluorescein angiography (FA), ultra-widefield imaging (UWF), and optical coherence tomography angiography (OCTA), with each imaging modality offering certain benefits over the others [24]. FA remains the gold standard in assessing the extent of ischemia. UWF imaging has allowed for the assessment of peripheral ischemia via FA. It is, however, OCTA that offers the best visualization of retinal vasculature with its noninvasive depth-resolved imaging and therefore has the potential to become a mainstay in the assessment of retinal ischemia [24].

Overview of Automated Image Analysis in DME

OCT plays a crucial role in the assessment of DME by providing detailed information about retinal thickness and fluid distribution [24]. Integrating AI algorithms has significantly advanced the field, automating the segmentation and analysis of OCT data. These algorithms focus on critical parameters such as central subfield thickness and intraretinal fluid (IRF) volume, enhancing the efficiency and accuracy of DME evaluation [25]. Deep learning techniques have brought about a revolutionary shift in automating major OCT parameters related to DME. These techniques, applied to quantify central subfield thickness and IRF volume, have shown notable advancements in accuracy and efficiency compared to traditional manual measurements [25]. AI algorithms designed for the automated detection of exudates and macula in DME contribute to streamlining the diagnostic process. Furthermore, this automated detection aids in grading the severity of the condition, providing valuable insights for clinicians managing DME cases [26]. Predicting treatment responses in patients with DME, particularly in the context of anti-VEGF therapy, has been a focus of exploration for AI techniques. These predictive models offer the potential for more informed decision-making in treatment planning and ongoing management [24]. The proposal of an end-to-end deep fusion model represents a cutting-edge approach to DME detection. Leveraging anatomical landmarks and operating on edge computing devices, this model aims to enhance the accuracy and efficiency of DME detection [26]. This holistic approach combines various deep-learning techniques for a more comprehensive assessment (Table 1).

**Table 1:** Difference between center-involved and non-center-involved DME DME: diabetic macular edema

Sr. no.	Major difference	Center-involved DME	Non-center-involved DME
1	Symptoms	It involves swelling in the fovea	It involves swelling in the macula but outside of the fovea
2	Quality of life	Swelling here will result in noticeable vision loss. This can affect the direct line of vision and make it hard to complete everyday tasks such as driving	The condition can still lead to vision loss, especially in the periphery. It does not affect the ability to focus on an object. This type of DME tends to have less effect on the quality of life compared to center-involved DME
3	Progresssion of condition	The area most responsible for your focused sight. Therefore, swelling in this region needs immediate attention	Most individuals with non-center-involved DME never progress to center-involved DME. People with non-center-involved DME may still have good visual acuity
4	Treatment and management regimens	For center-involved DME, intravitreal injections are the standard of care.	For non-center involved DME, a “wait-and-see” management strategy may be the best course of action

Machine Learning and Artificial Intelligence Applications

AI and machine learning (ML) have been increasingly applied in the management of DME. These technologies offer various benefits, including automated screening, precise evaluation, prognosis prediction, and DME follow-up monitoring [22,27]. One notable application is the development of AI tools for diagnosing DME and predicting treatment response using OCT. For instance, an AI algorithm achieved an accuracy of 87.5% in classifying normal and DME cases using OCT data [24]. Additionally, AI has been used to quantify fluid resolution and assess visual acuity gain in DME patients, providing valuable insights for treatment evaluation [27]. Furthermore, end-to-end deep fusion models have shown promise in detecting DME, with excellent performance in classifying the condition and visualizing anatomical landmarks [26]. These advancements demonstrate the potential of AI and ML in enhancing the efficiency and effectiveness of DME management, ultimately contributing to improved patient care and outcomes.

Advantages and Limitations of Automated Quantification

Automated quantification techniques present several advantages, including time and cost savings, rapid analysis and reporting, and heightened efficiency. In medical contexts, such as managing DME, automated quantification can assess disease activity, gauge treatment response, and predict treatment outcomes using AI and ML [22,28]. However, carefully considering the limitations associated with automated quantification is imperative. Technical challenges and constraints, such as reliance on technology and intricate systems, can potentially impact the reliability and accuracy of automated quantification [29]. In the medical realm, limitations may extend to infrastructure and space requirements, coupled with potential issues related to system failures and personnel shortages [30]. While automated quantification offers undeniable benefits in efficiency and swift analysis, a cautious approach is necessary to navigate this approach's technical challenges and limitations, particularly within medical applications.

Anti-VEGF intervention in DME

Role of VEGF in DME

The significance of VEGF in DME is profound, and anti-VEGF therapy has emerged as the preferred treatment strategy for its management. VEGF plays a critical role in the pathogenesis of DME, contributing to increased vascular permeability and edema. Noteworthy anti-VEGF drugs, including ranibizumab, aflibercept, bevacizumab, brolucizumab, and faricimab, have effectively reduced edema, improved vision, and prevented further visual loss in patients with DME [31,32]. However, it is crucial to acknowledge that a substantial proportion of DME patients may not respond adequately to anti-VEGF therapy, and some may continue to experience vision loss despite treatment efforts [33]. Various studies have identified predictive factors for treatment outcomes with anti-VEGF therapy, such as baseline vision, central macular thickness, and undertreatment [33]. This underscores the imperative for personalized treatment approaches and the exploration of alternative options, including intravitreal corticosteroids, laser photocoagulation, or surgical intervention, particularly for DME patients who exhibit resistance to anti-VEGF therapy [33]. Undoubtedly, anti-VEGF therapy plays a pivotal role in managing DME by explicitly targeting the underlying pathophysiology involving VEGF. While demonstrating benefits for many patients, a subset may not respond optimally to this treatment, emphasizing the ongoing need for further research and the development of personalized approaches to enhance outcomes for all individuals affected by DME.

Mechanism of Action of Anti-VEGF Agents

The mechanism of action of anti-VEGF agents is centered on inhibiting the activity of VEGF, a pivotal factor in angiogenesis and vascular permeability. VEGF plays a crucial role in promoting the mitosis of vascular endothelial cells, leading to angiogenesis and heightened vascular permeability. Anti-VEGF drugs, including bevacizumab, aflibercept, and ranibizumab, function by binding to soluble VEGF, thereby inhibiting its binding to VEGF receptors located on the surface of endothelial cells. This inhibition reduces angiogenesis and vascular permeability, ultimately mitigating macular edema and enhancing visual acuity, particularly in conditions like DR and ARMD [34,35]. Additionally, VEGF is recognized for its antiplatelet effects, primarily mediated through nitric oxide (NO) and prostacyclin (PGI2). Notably, bevacizumab may also directly affect platelet activation [36]. The effectiveness of anti-VEGF drugs varies, with different agents displaying distinct target selectivity, affinity, and potency in inhibiting the functional activity of proangiogenic factors [35]. In essence, anti-VEGF agents achieve their effects by impeding the activity of VEGF, consequently reducing angiogenesis and vascular permeability - critical processes in developing conditions like DME and ARMD.

Current Anti-VEGF Therapies and Their Efficacy

Anti-VEGF therapies have become widely utilized in the treatment of diverse ocular conditions, including ARMD, DME, and retinal vein occlusion (RVO). The primary anti-VEGF drugs employed in clinical practice are ranibizumab, aflibercept, and bevacizumab [35,37]. These drugs function by inhibiting the activity of VEGF, a pivotal factor in angiogenesis and vascular permeability, thereby reducing macular edema and enhancing visual acuity for patients with these conditions [35,38,37]. Research studies indicate that the efficacy of anti-VEGF drugs varies based on the specific drug, dosage, and therapeutic strategy employed [35,39]. For instance, aflibercept has demonstrated greater blocking potency than ranibizumab or bevacizumab [35]. However, a comprehensive analysis comparing the efficacy and safety of all currently utilized anti-VEGF therapies found that these treatments exhibited modest superiority over ranibizumab, aflibercept, and bevacizumab [35]. Despite these findings, controversies persist regarding the ideal drug and optimum therapeutic strategy. Challenges arise in acquiring comparative efficacy and safety profiles from current trials due to many therapeutic regimens involving anti-VEGF therapy, encompassing different drugs, dosages, and therapeutic approaches [39]. While anti-VEGF therapies effectively address various ocular conditions, the varying efficacy and safety profiles of different drugs, dosages, and therapeutic strategies highlight the need for further research to determine the optimal treatment approach tailored to individual patients. Addressing these considerations will contribute to refining the application of anti-VEGF therapies and improving outcomes in ophthalmic care (Table 2).

**Table 2:** Anti-VEGF drugs in the treatment of DME DME: diabetic macular edema; anti-VEGF: anti-vascular endothelial growth factor

Sr. no.	Drug	Doses	Therapeutic approaches
1	Bevacizumab	Available in 100 mg and 400 mg solution in 4 ml and 16 ml, respectively. The administration is via the intravenous route (IV). The first infusion is over 90 minutes and the subsequent infusion is over 60 minutes, if the first infusion is well tolerated.	Bevacizumab for the management of DME, focusing on the efficacy and duration of the clinical benefits of decreasing DME and the improvement of best-corrected visual acuity
2	Aflibercept	The recommended dose for aflibercept is 2 mg (0.05 ml) administered by intravitreal injection every four weeks	Aflibercept significantly improves functional and anatomical outcomes, and rapidly improves best-corrected visual acuity up to its peak; these results remain stable over time
3	Ranibizumab	Adult dose for macular edema DME and diabetic retinopathy with DME: 0.3 mg via intravitreal injection once a month	Ranibizumab is a medication used to manage and treat neovascular age-related macular degeneration, macular edema following retinal vein occlusion, DME, myopic choroidal neovascularization, and diabetic retinopathy

Integrating automated quantification into DME management

Potential Benefits of Automated Quantification in DME

Automated quantification introduces an objective and standardized approach to assessing intraretinal and subretinal fluid, enhancing the precision of evaluating disease severity and monitoring treatment response [40]. This method establishes a reliable foundation for clinical decision-making, providing consistency and objectivity in the interpretation of data. Implementing automated quantification techniques significantly improves the diagnosis and monitoring of DME. These techniques enhance diagnostic accuracy and streamline the monitoring process by enabling the automated detection and quantification of macular fluid through OCT. The automated nature of these approaches increases efficiency and ensures consistency in data interpretation over time [24]. Automated quantification is crucial in predicting treatment outcomes, particularly in anti-VEGF therapy. By leveraging quantitative data, these techniques assist in tailoring treatment strategies to individual patients and predicting the likely efficacy of interventions. This proactive approach supports optimized patient care and facilitates more informed treatment planning [22]. Automated quantification contributes significantly to our understanding of the anatomical alterations occurring in DME, particularly within the macular zone of the retina. Through comprehensive analysis facilitated by OCT, these techniques provide valuable insights into the structural changes associated with DME. This enhanced anatomical understanding aids in detecting and continuously monitoring the condition, contributing to a more nuanced comprehension of disease progression [41]. The clinical utility of automated analyses derived from spectral-domain OCT, facilitated by quantification techniques, is noteworthy. As valuable tools for assessing anatomical changes in DME patients, these analyses guide treatment decisions and provide a more in-depth understanding of the disease's progression. Integrating these quantitative findings into clinical practice contributes to personalized patient care and optimized treatment plans, enhancing overall clinical outcomes [40].

Improving Early Detection and Monitoring Through Automation

AI algorithms offer a more efficient and objective approach to detecting DME within retinal images than human graders. This heightened efficiency enables earlier detection of the condition and improves the overall monitoring process, potentially leading to more timely interventions and better patient outcomes [27]. One notable advantage of AI-driven techniques is their ability to reduce interobserver variability, showcasing similar sensitivity and higher specificity compared to human graders. This reduction in variability contributes to the reliability and consistency of diagnostic assessments for DME, emphasizing the potential for more accurate and standardized evaluations [27]. AI algorithms accurately quantify macular fluid volume (MFV) in patients with DME. This quantitative approach enhances the understanding of the disease and its progression, providing clinicians with precise insights into the extent of fluid accumulation within the macula. Detailed information can guide treatment decisions and interventions more effectively [25]. In monitoring treatment response, AI-based tools play a pivotal role by providing real-time data on the effectiveness of interventions. This functionality empowers clinicians to make informed decisions, facilitating timely treatment strategy adjustments and optimizing patient outcomes through personalized and responsive care [27]. The objective data generated by AI algorithms significantly enhances decision-making processes in managing DME. This includes determining the necessity for treatment and selecting the most appropriate intervention based on unbiased and thorough quantitative analyses. Incorporating AI-driven insights into decision-making processes contributes to the precision and effectiveness of clinical management for DME [42].

Challenges in Implementing Automated Quantification in Clinical Practice

Implementing automated quantification techniques in clinical practice hinges on establishing standardized processes. Such standardization is necessary for variability in results to ensure widespread adoption, underscoring the need for uniform protocols and procedures to ensure consistency in applying these techniques [43]. Thorough validation is critical for automated quantification techniques to guarantee accuracy and reliability in clinical settings. However, this validation process can be resource-intensive and time-consuming, posing a challenge to efficiently integrating these technologies into routine clinical workflows [44]. Rigorous validation procedures are essential for building confidence in the credibility and effectiveness of automated quantification methods. The interpretability of results generated by automated quantification techniques, especially those involving complex algorithms, presents a challenge for clinicians. Additional training may be necessary to enhance healthcare professionals' understanding and interpretation of these results, promoting their effective integration into clinical decision-making processes [45]. The quality of data utilized for automated quantification holds significant sway over the accuracy of the results. To maintain high precision, meticulous data acquisition, storage, and processing control is imperative. Attending to data quality ensures avoiding errors or biases that could compromise the reliability of automated quantification outcomes [44]. Using automated quantification techniques in clinical practice introduces ethical and legal considerations that demand careful attention. Patient privacy, data ownership, and liability must be addressed to ensure these techniques' safe and ethical application [43]. Establishing robust frameworks and guidelines is essential to navigate the ethical and legal landscape associated with the deployment of automated quantification in healthcare settings.

Future directions in DME management

Emerging Technologies in DME Diagnosis and Management

Cutting-edge technologies in the diagnosis and management of DME encompass innovative treatment modalities, genetic applications, anti-VEGF therapy, and personalized treatment recommendations [46]. One notable avenue involves the non-invasive delivery of medication through eye drops, showcasing significant improvements in visual acuity, particularly in individuals undergoing Oculis treatment [47]. Vantage Biosciences is pioneering the development of an oral drug targeting DR and DME. This drug aims to inhibit AOC-3 (amine oxidase copper-containing 3), a protein integral to the inflammatory response in DME [47]. Research also indicates that drugs binding to soluble VEGF can repair the blood-retinal barrier, alleviate macular edema, and enhance vision for most DME patients [41]. Another emerging technology involves automated quantifying of macular fluid in retinal diseases and their response to anti-VEGF therapy using AI, presenting a promising avenue in DME management [22]. These advancing DME diagnosis and management technologies are geared toward enhancing patient outcomes and averting vision loss.

Personalized Medicine Approaches in DME

Personalized medicine approaches are gaining prominence in managing DME, emphasizing customized treatments based on individual patient characteristics. This recognition stems from the understanding that DME can manifest with diverse underlying causes and mechanisms across patients, resulting in varying responses to standard therapies, such as anti-VEGF injections [48]. An essential facet of personalized treatment involves pinpointing each patient's primary cause of DME. This cause can range from being purely VEGF-driven to inflammation-driven or a combination of both. Tailoring the treatment approach, such as adjusting the dosage of anti-VEGF therapy based on the underlying cause, is crucial for optimizing outcomes [48]. Beyond conventional anti-VEGF injections, emerging personalized medicine approaches in DME management encompass the development of non-invasive treatment options. This includes eye drops and oral drugs targeting specific inflammatory pathways involved in DME. Research is actively exploring the potential of non-invasive delivery methods, such as eye drops and oral drugs designed to obstruct proteins integral to the inflammatory response in DME [47]. Moreover, the application of AI in precision medicine for DME represents a burgeoning area of research. Advanced technologies are employed to analyze retinal morphology and predict treatment outcomes, enabling more personalized and effective treatment strategies for DME patients [22,41]. In essence, personalized medicine approaches in DME management strive to enhance treatment outcomes by tailoring interventions to the unique characteristics of each patient's condition. This personalized approach holds promise for better managing this vision-threatening complication of diabetes [47,48].

Combining Automated Quantification With Novel Interventions

In DME management, cutting-edge technologies are reshaping treatment paradigms. AI and ML have taken center stage, showcasing the development of algorithms adept at predicting treatment outcomes post anti-VEGF injections. These AI algorithms boast an impressive accuracy rate of up to 81%, particularly in forecasting post-treatment central foveal thickness (CFT) and best-corrected visual acuity (BCVA) [49]. Furthermore, AI plays a pivotal role in streamlining the quantification of macular fluid in retinal diseases, offering valuable insights into their responsiveness to anti-VEGF therapy [25]. Novel approaches in DME management extend to non-invasive treatments, notably the creation of eye drops delivering medication. This innovative avenue has significantly improved visual acuity among patients undergoing this treatment modality [48]. On the pharmaceutical front, Vantage Biosciences is pioneering the development of an oral drug tailored for DR and DME. This oral medication specifically targets AOC-3, a pivotal protein in the inflammatory response within the context of DME [48]. Recognizing the diverse responses among DME patients, there is an escalating call for greater personalization in treatment strategies. Standard therapies like anti-VEGF injections yield variable responses, ranging from cases purely driven by VEGF to those combining VEGF and inflammation-driven factors and others primarily influenced by inflammatory responses [48]. In precision medicine, integrating "omics" information, including pharmacogenomics and immune therapies, emerges as a promising strategy. This approach empowers clinicians to stratify patients based on individual risk profiles, facilitating the selection of optimal follow-up and treatment strategies. Precision medicine, guided by comprehensive "omics" data, holds the potential to tailor interventions to the unique characteristics of each patient [50].

Case studies and clinical trials

Overview of Recent Clinical Trials in DME

Recent clinical trials in DME have introduced significant investigations to advance treatment options. One notable randomized clinical trial, part of the KINGFISHER study, delved into assessing the efficacy and safety of brolucizumab versus aflibercept, with both administered every four weeks [51]. Although the study detected no clinically meaningful difference between the brolucizumab and aflibercept arms, some superior anatomic improvements were observed in the brolucizumab arm [51]. In another clinical trial, a cohort of 442 DME patients is undergoing treatment with either PRO-169 (bevacizumab) or Lucentis® (ranibizumab) [52]. The American Academy of Ophthalmology Annual Meeting in San Francisco became a platform for discussing advancements in DME treatment, featuring new data on recently approved treatments and papers exploring the underrepresentation of diverse populations in DME-related clinical trials [53]. A comprehensive review of clinical trials focused on DME sheds light on the safety and efficacy of pharmacological agents, notably anti-VEGF therapy. This review also offers an update on ongoing clinical trials to advance DME treatment options [54].

Success Stories and Challenges in Implementing Innovations

The prevalence of DR and DME presents substantial global public health challenges, with a projected increase in affected individuals in the foreseeable future. Encouragingly, progress has been made in combating these conditions, which is attributable to enhanced systemic management, expanded DR screening initiatives, and improved accessibility to advanced ocular treatments, including laser photocoagulation and intravitreal therapy [55]. In certain regions, the positive impact of these concerted efforts is evident, as diabetic retinopathy/maculopathy is no longer the primary cause of certifiable blindness [55]. Despite these advancements, the pervasive nature of the problem and the global implications of diabetes and DR underscore the ongoing need for comprehensive, systemwide strategies to tackle these challenges effectively [55]. In clinical trials, continuous research endeavors are underway to assess the efficacy and safety of diverse treatment options for DME. One notable ongoing clinical trial is actively recruiting 442 patients with DME, offering them treatment options with either PRO-169 (bevacizumab) or Lucentis® (ranibizumab) [52]. These trials exemplify the sustained commitment to developing and enhancing treatments for DME, with the overarching goal of meeting the unmet medical needs of individuals grappling with this condition.

Promising Outcomes and Areas of Improvement

Recent advancements in treating DME have showcased promising outcomes, revealing both successes and areas of refinement. The PHOTON trial notably underscored the efficacy and safety of aflibercept in treating DME, positioning it as a potentially impactful treatment option [56]. Additionally, higher-dose formulations of aflibercept and the bispecific molecule farcical have exhibited promising results, demonstrating comparable or enhanced visual acuity gains and extended treatment intervals. These developments hold significant potential for enhancing the quality of life for patients managing DME [53]. Moreover, research indicates that drugs targeting soluble VEGF can be crucial in repairing the blood-retinal barrier, alleviating macular edema, and improving vision for individuals with DME [41]. Nevertheless, real-world outcomes may need to align more closely with those observed in randomized clinical trials. Efforts are ongoing to ascertain the optimal treatment regimens tailored to individual patients, emphasizing the necessity for continued research in this domain [41]. While considerable strides have been made in the battle against DME, the escalating global prevalence of diabetes and DME demands sustained efforts. Ongoing initiatives are essential to furnish updated and precise epidemiologic data, fostering the development of comprehensive, systemwide strategies to address these challenges effectively [55]. The recent progress in DME treatment presents encouraging outcomes, promising enhanced patient care and improved quality of life. Nevertheless, the imperative for continuous research and inclusive clinical trials remains to propel the field forward and contend with the mounting prevalence of DME worldwide.

## Conclusions

In conclusion, this comprehensive review illuminates the transformative landscape of DME management, showcasing the potential of recent innovations. Automated quantification techniques and advancements in anti-VEGF interventions offer a promising trajectory toward more precise and effective diagnostic and therapeutic strategies for DME. The summary of key findings underscores the progress made in understanding the pathophysiology of DME and the positive impact of emerging technologies. Looking ahead, the implications for the future of DME management are profound, with the prospect of personalized medicine and improved treatment outcomes. However, recognizing the complexities of DME, a resounding call to action resonates for further research and collaboration. Continuous efforts are needed to refine automated quantification tools, assess long-term safety and efficacy, and seamlessly integrate these innovations into clinical practice. Collaboration across disciplines and stakeholders is essential to drive collective progress, address emerging challenges, and propel DME management toward a future marked by precision medicine and enhanced patient care.
